# Effectiveness of a Web-Based SUpport PRogram (SUPR) for Hearing Aid Users Aged 50+: Two-Arm, Cluster Randomized Controlled Trial

**DOI:** 10.2196/17927

**Published:** 2020-09-22

**Authors:** Janine FJ Meijerink, Marieke Pronk, Birgit I Lissenberg-Witte, Vera Jansen, Sophia E Kramer

**Affiliations:** 1 Otolaryngology—Head and Neck Surgery, Ear and Hearing Amsterdam Public Health research institute Amsterdam UMC, Vrije Universiteit Amsterdam Amsterdam Netherlands; 2 Epidemiology and Data Science Amsterdam UMC Vrije Universiteit Amsterdam Amsterdam Netherlands; 3 Schoonenberg HoorSupport Dordrecht Netherlands

**Keywords:** hearing loss, hearing aids, auditory rehabilitation, self-management, communication programs, internet, hearing aid dispensing practice, randomized controlled trial

## Abstract

**Background:**

Hearing aid (HA) use is known to improve health outcomes for people with hearing loss. Despite that, HA use is suboptimal, and communication issues and hearing-related activity limitations and participation restrictions often remain. Web-based self-management communication programs may support people with hearing loss to effectively self-manage the impact of hearing loss in their daily lives.

**Objective:**

The goal of the research is to examine the short- and long-term effects of a web-based self-management SUpport PRogram (SUPR) on communication strategy use (primary outcome) and a range of secondary outcomes for HA users aged 50 years and older.

**Methods:**

Clients of 36 HA dispensing practices were randomized to SUPR (SUPR recipients; n=180 HA users) and 34 to care as usual (controls; n=163 HA users). SUPR recipients received a practical support booklet and online materials delivered via email over the course of their 6-month HA rehabilitation trajectory. They were encouraged to appoint a communication partner and were offered optional email contact with the HA dispensing practice. The online materials included 3 instruction videos on HA handling, 5 videos on communication strategies, and 3 testimonial videos. Care as usual included a HA fitting rehabilitation trajectory only. Measurements were carried out at baseline, immediately postintervention, 6 months postintervention, and 12 months postintervention. The primary outcome measure was self-reported use of communication strategies (3 subscales of the Communication Profile for the Hearing Impaired [CPHI]). Secondary outcome measures included self-reported personal adjustment to hearing loss (CPHI); use, satisfaction and benefit of HAs and SUPR (use questionnaire; International Outcome Inventory for Hearing Aids [IOI-HA], Alternative Interventions [IOI-AI]); recommendation of HA dispensing services; self-efficacy for HA handling (Measure of Audiologic Rehabilitation Self-Efficacy for Hearing Aids [MARS-HA]); readiness to act on hearing loss (University of Rhode Island Change Assessment adapted for hearing loss [URICA-HL]); and hearing disability (Amsterdam Inventory for Auditory Disability and Handicap [AIADH]).

**Results:**

Linear mixed model analyses (intention to treat) showed no significant differences between the SUPR and control group in the course of communication strategy use (CPHI). Immediately postintervention, SUPR recipients showed significantly higher self-efficacy for advanced HA handling than the controls, which was sustained at 12 months (MARS-HA; mean difference immediately postintervention: 5.3, 95% CI 0.3 to 10.4; *P*=.04). Also, SUPR recipients showed significantly greater HA satisfaction than controls immediately postintervention (IOI-HA; 0.3, 95% CI 0.09 to 0.5; *P*=.006), which was sustained at 12 months, and significantly greater HA use than the controls immediately postintervention (IOI-HA; 0.3, 95% CI 0.02 to 0.5; *P*=.03), which was not sustained at 12 months.

**Conclusions:**

This study provides ground to recommend adding SUPR to standard HA dispensing care, as long-term, modest improvements in HA outcomes were observed. Further research is needed to evaluate what adjustments to SUPR are needed to establish long-term effectiveness on outcomes in the psychosocial domain.

**Trial Registration:**

ISRCTN77340339; http://www.isrctn.com/ISRCTN77340339

**International Registered Report Identifier (IRRID):**

RR2-10.1136/bmjopen-2016-015012

## Introduction

Hearing loss is highly prevalent among older adults. Approximately one-third of adults aged 65 years or over have a disabling hearing loss [1]. Importantly, hearing loss is known to be associated with various negative health outcomes including falls [2], loneliness and depression [3-5], incident dementia [6,7], and even mortality [8,9]. The main clinical rehabilitation option for people with hearing loss is the provision of hearing aids (HAs) [10]. Although there is evidence that HA use can reverse negative effects [11-15], a substantial proportion of HA owners, estimated at 3% to 24%, never wear them [16-21]. Others still experience problems in their daily lives when wearing them [22]. Underlying reasons for such negative experiences include limited social support [23], low acceptance of hearing loss [24], problems with handling the devices [24-26], and poor use of supporting communication skills [22] (ie, strategies to enhance communication such as speechreading and reducing distance to the speaker). These reasons suggest that hearing rehabilitation should not be limited to HA fitting alone but also support users in addressing their residual hearing-related activity limitations and participation restrictions, while taking into account their personal and external contextual factors [27,28].

There are several educational communication programs providing support in these domains. They teach HA users knowledge and skills to effectively self-manage the multidimensional impact of hearing loss—for instance, by providing information about hearing, teaching communication strategies, or counseling to support better coping with the consequences of hearing loss [22,29-34]. While some programs proved to be effective in terms of HA benefit and use of communication strategies, their long-term effects are largely unknown [10]. Moreover, none of them were widely implemented in hearing health care practices [35], mainly because of limited resources [36] and high costs [28]. Delivery of communication programs via eHealth holds great promise because it potentially allows for providing services at the intensity that the patient prefers, automatized delivery with limited efforts for health care professionals, and wide reach, thereby ultimately improving (cost-) effectiveness and access to hearing care [37].

An example of a recently developed effective e-support program is the multimedia educational program c2HEAR [32]. This is based on reusable learning objects, which are short interactive videos covering information on HA handling, communication strategies, and adaptation to wearing HAs. They are delivered through DVD for television, over the internet, or on the PC [32]. The program was tested in a sample of first-time HA users attending the Nottingham Audiology Service (part of public hearing care in the United Kingdom) and appeared successful in improving HA use and practical HA skills. Another example is the effective web-based program by Malmberg et al [31], who evaluated it in Swedish general clinical practices among experienced HA users. Their program included online reading material combined with home training on hearing, HAs, and communication strategies and an online peer discussion forum. The program yielded improved communication skills [31]. It is crucial to test web-based support programs in these types of real-world, clinical care settings that are accessed by most of the adults with hearing loss seeking hearing care.

We recently developed a web-based self-management educational support program for adult HA users and their communication partners called SUPR (short for SUpport PRogram), to be offered within the HA dispensing care setting as an addition to a regular HA fitting trajectory. The home education program by Kramer et al [33], an intervention successful in improving communication strategy use and quality of life at 6-month follow-up, lies at the foundation of SUPR. The aim of our study was to evaluate the short term (ie, immediately postintervention) and the long term (ie, up to 12 months postintervention) effectiveness of SUPR on the use of communication strategies and a range of secondary outcomes compared with HA fitting alone. For that purpose, we performed a large-scale cluster randomized controlled trial (cRCT) including clients of one of the major HA dispenser chains in the Netherlands (Schoonenberg HoorSupport). The design of the cRCT has been described elsewhere [38]. The main hypothesis was that HA users receiving SUPR would improve their use of communication strategies (primary outcome) and improve several secondary outcomes (see Methods), while HA users receiving HA fitting without additional support would not. Furthermore, we hypothesized that the effects would be larger for first-time than for experienced HA users as was also observed for the home education program [33].

## Methods

### Study Design

The study was conducted and reported according to the Consolidated Standards of Reporting Trials (CONSORT) statement for cRCTs [39]. This study had a 2-arm cRCT design with the HA dispensing practice (henceforth: practice) as the unit of allocation. Cluster randomization was preferred over individual randomization to minimize the risk of contamination. The sample size calculation had indicated that 70 practices should participate in the study. Participating practices were prestratified by level of urbanization (located either in relatively rural or urban areas). Within both strata, a statistician randomized practices to either the intervention or control arm. The randomization sequence was generated by the statistical software R (R Foundation on Statistical Computing) with random permutation in blocks of size 4 with a fixed seed. Thirty-four practices were allocated to the control arm and 36 to the intervention arm.

Participants, HA dispensers (henceforth: dispensers), and researchers were aware of the practices’ and participants’ trial arm allocation. Participants and dispensers could not be blinded due to the nature of the intervention. Researchers could not be blinded because they administered all questionnaires to the participants, including the International Outcome Inventory (IOI), which revealed trial arm allocation. Additionally, researchers actively monitored the uptake of SUPR for the purposes of the process evaluation (submitted), also revealing arm allocation. The study was approved by the Dutch Institutional Review Board of the VU University Medical Center Amsterdam (IRB00002991; FWA number: FWA00017598) and registered [ISRCTN77340339].

### Setting and Participants

Participants were recruited between February and September 2016 by the dispensers or their supporting staff of the participating practices. They informed each potentially eligible client about the study when clients were about to enter an HA trial period. Interested clients received an information package. The package contained information on the general study aims and group allocation (SUPR or control). No details about the SUPR program other than that it is a support program aimed at improving communication was provided at that point to ensure that clients would not ask or seek information about SUPR (thereby preventing contamination of the control group). Clients could sign up for the study via an online webpage and provided their consent there.

Eligible participants were Dutch speaking adults aged 50 years or over who had decided to purchase one or two HAs, had access to a device with internet connection, and were owner of an email account for the total duration of the study. These purchased HAs were the first ones for the first-time HA users and replacement HAs for the experienced users. We excluded participants who received additional care via an audiology clinic because this type of care might interfere with that provided in SUPR. We also excluded participants who purchased HAs to suppress tinnitus because tinnitus management was not included in SUPR.

### Intervention

#### Control

Practices allocated to the control arm offered care as usual (HA fitting rehabilitation trajectory only). Briefly, the care usually provided included 4 face-to-face appointments with the dispenser. During the first appointment, a screening pure-tone audiogram was administered, and the client’s goals and preferences were discussed. Clients were also asked to assign a communication partner and bring them along to future appointments. The communication partner could be any person the client communicated with on a regular basis (ie, a partner, child, neighbor, or caregiver). During the second appointment, additional hearing tests were performed. The HAs were then fitted immediately (if available) or at the third appointment. Clients were subsequently able to try out their HAs during a period of around 4 weeks in order to decide whether to purchase them. During a fourth appointment, the client’s experiences and decision to purchase the HAs were discussed. Follow-up appointments were scheduled if needed. In addition, clients were able to visit the practice for small problems (ie, HA repairs) every working day during a service hour (4:00 to 5:00 pm) and contact the practice via telephone or an online form.

#### SUPR

The intervention group received the SUPR program as an addition to their HA fitting trajectory. A full description of the development of SUPR is reported elsewhere [40].

SUPR comprised 4 main elements: practical support booklet, emails, optional contact with the practice customer contact service, and involvement of a communication partner.

The practical support booklet, which clients received during their first appointment, contained tips and information on HAs, hearing loss, and communication strategies. Clients were instructed to write down their specific needs and goals they wished to reach with their HAs. These were discussed with the dispenser during follow-up appointments and were used for further refinement of the HA fitting.

**Figure 1:**
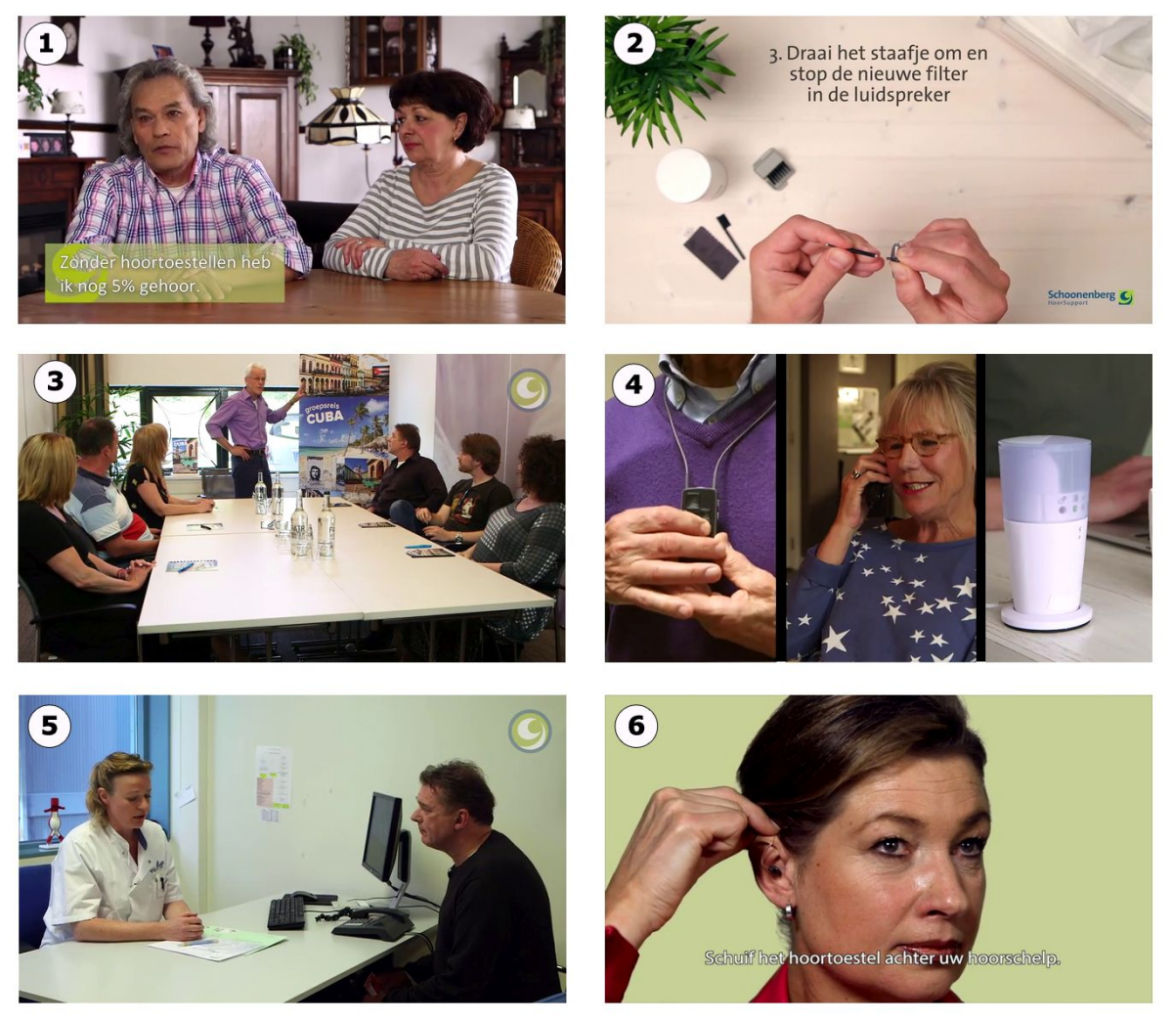
Stills of the SUPR educational videos: (1) testimonial video in which peers talk about their experiences with hearing loss; (2) instruction video on cleaning and maintenance of hearing aids; (3) video on how to apply effective communication strategies in a group setting; (4) instruction video on using assistive listening devices; (5) video on how to apply effective communication strategies in a one-to-one conversation; and (6) instruction video on how to insert hearing aids.

A total of 17 emails were delivered over 6 months (approximately one email every 2 weeks). The first email was sent on the day the client started their HA trial period. The last email was sent approximately 6 months after the client had purchased the HA. Eleven emails contained links to educational videos, including 3 instruction videos on HA handling (eg, how to insert their specific type of HA), 5 videos on communication strategies and personal adjustment (eg, illustrating how to apply communication strategies in a birthday party setting), and 3 testimonial videos in which peers shared their experiences. Still photos from the different educational videos are shown in Figure 1. We are not able to provide a direct link to one of the videos in this manuscript since it is commercially sensitive information. The remaining 6 emails contained links to written communication strategy tips and information on HA maintenance. Two offered the client the option to contact the practice. The emails were delivered via a fully automated system. Access was free of charge. The intervention could be used ad libitum, and no reminders or prompts were used to encourage clients to watch the videos. The researchers were able to check if participants had clicked on the email links to the educational videos.

Clients were invited to share their opinion regarding their HAs and SUPR by replying to emails 12 and 16 for optional contact with the practice customer contact service.

Clients were advised to assign a communication partner and involve them during the HA fitting trajectory and SUPR. The communication partner could sign up to receive the SUPR emails on behalf of their loved one if they did not own an email account. Clients were encouraged to watch the videos together with their communication partner. Communication partners were encouraged to write down their goals and experiences with the HAs of their loved ones in the practical support booklet.

### Outcomes

All outcomes were collected via online questionnaires at baseline (t0: before the HA trial period), immediately postintervention (t1: 6 months after the HA purchase), 6 months postintervention (t2), and 12 months postintervention (t3). The outcomes were measured at all time points except for the ones that did not apply at t0 (ie, HA use—participants had not yet obtained their HAs).

#### Primary Outcome

The primary outcome was use of communication strategies as measured by 3 subscales of the Dutch Communication Profile for the Hearing Impaired (CPHI; maladaptive behaviors, verbal strategies, nonverbal strategies) [41,42]. Scores range from 1 to 5. The Dutch CPHI has a clear factor structure, and the subscales have good reported internal consistency (Cronbach alpha coefficients, henceforth alphas, between .81 and .86) [43].

#### Secondary Outcomes

Table 1 presents an overview of all secondary outcomes and the time points at which they were measured.

**Table 1:** Secondary outcomes measures (ranges) and time points.

Outcome measure		t0	t1	t2	t3
**Personal adjustment to hearing loss (CPHI^a^)**					
	Self-acceptance (1-5)	x	x	x	x
	Acceptance of loss (1-5)	x	x	x	x
	Stress and withdrawal (1-5)	x	x	x	x
Emotional response (HHDI^b^; 0-4)		x	x	x	x
**Self-efficacy for HA^c^ handling (MARS-HA^d^)**					
	Basic (0-100)		x	x	x
	Advanced (0-100)		x	x	x
HA use (IOI-HA^e^; 1-5)			x	x	x
HA pattern (use questionnaire; 1-5)			x	x	x
**HA rehabilitation outcomes (IOI-HA)**					
	Satisfaction (1-5)		x	x	x
	Quality of life (1-5)		x	x	x
**HA rehabilitation outcomes and SUPR rehabilitation outcomes (IOI-HA and IOI-AI^f^)**					
	Benefit (1-5)		x	x	x
	Residual activity limitations (1-5)		x	x	x
	Satisfaction (1-5)		x	x	x
	Residual participation restrictions (1-5)		x	x	x
	Impact on others (1-5)		x	x	x
	Quality of life (1-5)		x	x	x
Recommendation of the services of the HA dispensing practice (1-10)		x	x	x	x
**Readiness to act on hearing loss (URICA-HL^g^)**					
	Precontemplation (problem denial; 1-5)	x	x	x	x
	Contemplation (problem awareness; 1-5)	x	x	x	x
	Preparation (information seeking and need for professional guidance; 1-5)	x	x	x	x
	Action (healthy behavior acquisition or modification; 1-5)	x	x	x	x
	Maintenance (sustained healthy behavior; 1-5)		x	x	x
	Readiness (16-80)		x	x	x
	Committed action (5-37)	x	x	x	x
**Self-reported hearing disability (AIADH^h^)**					
	Distinction of sounds (0-24)	x	x	x	x
	Auditory localization (0-15)	x	x	x	x
	Intelligibility in noise (0-15)	x	x	x	x
	Intelligibility in quiet (0-15)	x	x	x	x
	Detection of sounds (0-15)	x	x	x	x

^a^CPHI: Communication Profile for the Hearing Impaired.

^b^HHDI: Hearing Handicap and Disability Inventory.

^c^HA: hearing aids.

^d^MARS-HA: Measure of Audiologic Rehabilitation Self-Efficacy for Hearing Aids.

^e^IOI-HA: International Outcome Inventory for Hearing Aids.

^f^IOI-AI: International Outcome Inventory for Alternative Interventions.

^g^URICA-HL: University of Rhode Island Change Assessment adapted for hearing loss.

^h^AIADH: Amsterdam Inventory for Auditory Disability and Handicap.

Psychosocial measures were assessed in two ways. First, personal adjustment to hearing loss was measured using 3 other subscales of the Dutch CPHI [41,42] (Table 1). The scales have good reported internal consistency (all alphas >.85) [43]. Second, the section emotional response of the Hearing Handicap and Disability Inventory (HHDI) was used [44]. As no psychometric information was available for this scale, we investigated the internal consistency in the current data set, which appeared good (alpha=.80).

Self-efficacy for HA handling was measured using the scales of the Measure of Audiologic Rehabilitation Self-Efficacy for Hearing Aids (MARS-HA; Table 1) [45]. The MARS-HA has a clear factor structure, and the scales showed reasonable to good internal consistency (alphas .67 to .88) [45].

HA use was measured in two ways. First we used the use item of the validated IOI-HA scale [46,47]. Each item (including the use item) can be used separately [47]. Second, we used one item of the use questionnaire as developed by Laplante-Lévesque et al [48].

HA rehabilitation outcomes were measured using 2 items of the IOI-HA [46,47] for both the control and SUPR group (Table 1). The remaining rehabilitation outcomes were measured using 6 items of the IOI-HA for the control group and the IOI for Alternative Interventions (IOI-AI) for the SUPR group (Table 1) [49]. Comparing the item scores between the groups allowed us to compare rehabilitation outcomes (ie, comparing HA outcomes for the controls and SUPR rehabilitation outcomes for the SUPR group). The IOI-AI has good psychometric properties and individual items can be used [47,50] (and compared to their counterpart items of the IOI-HA) [49].

Recommendation of the services of the practice was measured using the question: “How likely is it that you would recommend the services of the practice to other people (family, friends, colleagues)?”

Self-reported hearing disability was measured using the 5 subscales of the Amsterdam Inventory for Auditory Disability and Handicap (AIADH) [51,52] (Table 1). The AIADH has a clear factor structure, and each subscale has good reported internal consistency (alphas .75 to .91) [52].

The Dutch version of the University of Rhode Island Change Assessment adapted for hearing loss (URICA-HL) [53] was used to assess participant readiness on 5 stages of change (Table 1). In addition to the stage scores, the composite readiness score (contemplation + action + maintenance scores – precontemplation) and the committed action score (action-contemplation) were calculated [54]. Laplante-Lévesque et al [54] found a clear factor structure for the stages and reported good internal consistency (all alphas >.76).

### Statistical Analysis

#### Sample Size

The required sample size was calculated separately for first-time and experienced HA users. To detect a clinically meaningful effect of 0.67 [55] on the communication strategies CPHI subscale maladaptive behaviors (ie, the subscale with the smallest clinically meaningful effect) in first-time HA users (power 80%, significance level 5%, intracluster correlation coefficient [ICC] .01), two first-time HA users from each of the 70 practices would be needed in the analysis. To detect a clinically meaningful effect of 0.4 in experienced HA users (power 80%, significance level 5%, ICC .01), 3 clients from each practice would be needed in the analysis. As we anticipated a 20% loss to follow-up and a 30% recruitment rate, 4 first-time and 5 experienced clients were aimed to be recruited per practice.

#### Data Analysis

A statistical analysis plan was written and agreed upon before data analysis was started. Note that although we originally planned to use the overall summed score of the AIADH [38], it was agreed that the 5 subscale scores would provide a more detailed insight. In addition, measuring HA use objectively via HA data logging was not feasible as it turned out that these data were not collected as part of standard procedures in the practices.

Independent samples *t* tests, Mann-Whitney *U* tests, and chi-square tests were used to determine whether client characteristics were similarly distributed across the experimental conditions. Linear mixed models with fixed effects for group, time, and their 2-way interaction (ie, time*group), and random intercepts for subjects and practices were used to test differences in the course of the outcomes (t0 to t3 or t1 to t3) between the groups. Post hoc analyses based on the estimated fixed effects were carried out in case significant group differences in the course in outcomes were found to assess at which time points these occurred. For outcomes assessed at t1 to t3, we also examined whether there was a significant difference between the groups at t1 to determine the immediate posttreatment effect. To illustrate, in case of a statistically significant group difference at t1, a nonstatistically significant interaction term would indicate that this group difference was maintained at t2 and t3. In contrast, a statistically significant interaction term would indicate that this group difference changed (ie, either disappeared or worsened). Nonnormality was checked for all outcomes and data were transformed when necessary. Potential confounders (client characteristics) were examined for all outcomes and added as (fixed) covariates to the model in case they were differently distributed (*P*<.05) across groups at t0. Subgroup differences, using 3-way (time*group*type of client) interaction terms, were performed to check whether any intervention effects differed between first-time and experienced clients.

Main analyses were performed on the principle of intention to treat (ITT). A per protocol analysis was additionally performed including SUPR recipients who had clicked through to a video on communication strategies and personal adjustment from at least two emails and controls who did not receive any SUPR emails. Bonferroni corrections for multiple testing were applied for the primary outcome (3 subscales) and for the post hoc analyses (3 follow-up measurements), such that a *P*<.016 (0.05/3) was considered to indicate a statistically significant group difference. For all secondary outcomes, a *P* value of <.05 was considered statistically significant. In case significant group differences were found at t1, t2, or t3, mean differences between the mean values in the SUPR and control group were reported along with their 95% confidence interval and *P* value. Because a linear mixed model gives unbiased results in the presence of missing data, imputation of missing outcomes was not considered. Thus, clients were included in the analyses if they had provided data at one time point at least. Analyses were carried out using SPSS Statistics version 26 (IBM Corp).

## Results

### Study Population

**Figure 2:**
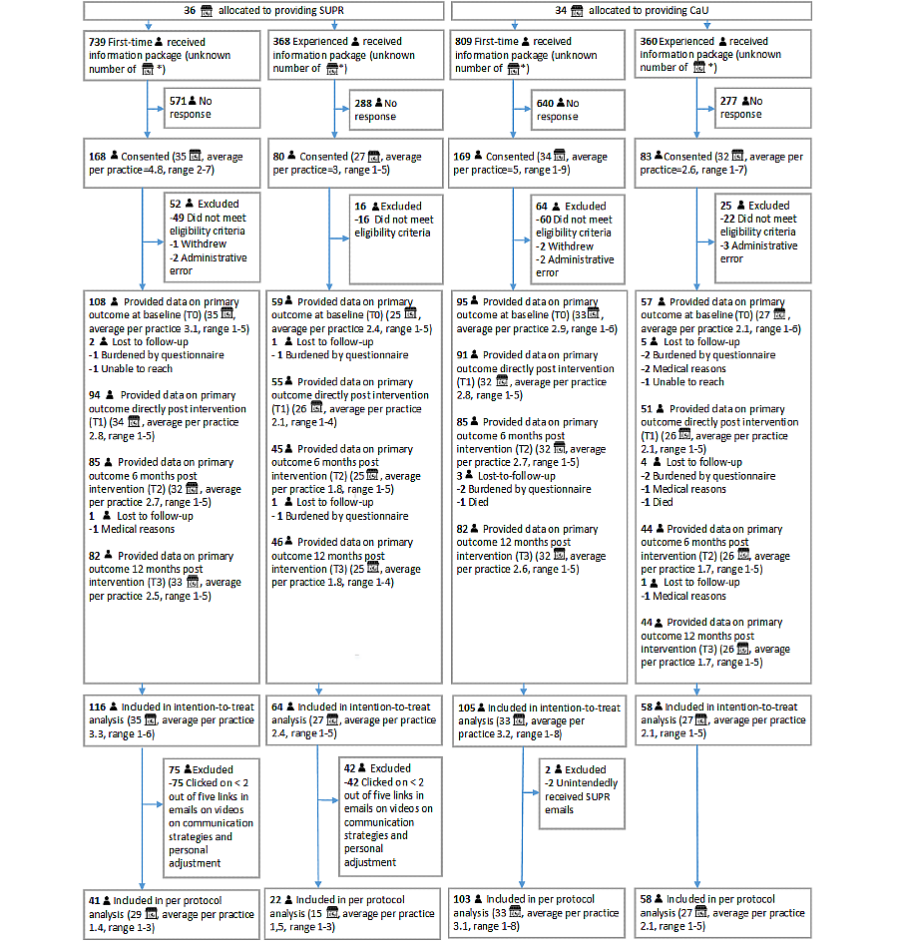
Flow of participants through the study. *Per practice, an unknown number of respondents was invited to participate due to some dispensers' noncompliance with the protocol to report this. The number of invited clients is an estimation based on the reported numbers of invited clients of the practices complying with the protocol to report this.

Figure 2 displays the flow of first-time and experienced clients through the study. Between February and September 2016, 1107 (739 first-time and 368 experienced) clients in the intervention arm and 1169 (809 first time and 360 experienced) clients in the control arm were invited to participate in the study. The number of invited clients is an estimation based on the reported numbers of invited clients of the practices complying with the protocol to report this.

In total, 248 clients (across 35 practices in the SUPR arm) and 252 clients (34 practices in the control arm) enrolled for the study and consented to participate. Of these, 180 and 163 clients were included in the ITT analysis, respectively. Loss to follow-up was 2.8% (5/180) in the SUPR arm and 8.0% in the control arm (13/163). Sixty-three clients in the SUPR arm and 161 controls were included in the per protocol analysis.

Table 2 presents the baseline characteristics of the participants. The number of participants in each practice ranged from 1 to 11 (mean 5). Of the total sample, 60% (206/343) were male (mean age 68.1 [SD 8.5] years), and mean pure-tone hearing loss was 43.7 (SD 11.1) decibels Hearing Level. The characteristics for SUPR and control participants were similar, as were the outcomes at baseline (*P*>.05), indicating that correction in the analyses due to significant group differences was not required.

**Table 2:** Baseline characteristics.

Characteristics		SUPR group	Control group
**Sex**		n=180	n=163
	Male, n (%)	108 (60)	98 (60)
Age in years, mean (SD)		68.1 (8.4)	68.2 (8.7)
**Marital status, n (%)**		n=177	n=158
	Married	130 (73)	111 (70)
	Cohabiting	9 (5)	8 (5)
	Widowed	24 (14)	14 (9)
	Divorced	7 (4)	16 (10)
	Single, never married	7 (4)	9 (6)
**Living situation, n (%)**		n=177	n=158
	Living together with other people	144 (81)	122 (77)
	Living alone	33 (19)	36 (23)
**Educational level, n (%)**		n=177	n=157
	Low	38 (22)	28 (18)
	Middle	123 (70)	110 (70)
	High	16 (9)	19 (12)
**Paid job, n (%)**		n=177	n=158
	Yes	39 (22)	38 (24)
	No	138 (78)	120 (76)
**Country of birth, n (%)**		n=177	n=158
	The Netherlands	162 (92)	149 (94)
	Other	15 (9)	9 (6)
Better ear average hearing loss in dB HL^a^, mean (SD)		43 (11.7)	44.5 (10.5)
Bilaterally fitted hearing aids (ie, two ears)^b^		132 (89)	126 (89)

^a^dB HL: decibels hearing level averaged across 1, 2, and 4 kilohertz.

^b^Measured via the t1 questionnaire (n=291).

### Primary Outcomes

#### Intention to Treat and Per Protocol Analysis

The ITT analysis showed no statistically significant (*P*≥.016) group differences in the course of communication strategy use (Table 3). The per protocol analysis showed no significant (*P*≥.016) group differences either (Table 3). There were no differences in effects between first-time and experienced clients.

**Table 3:** Descriptive statistics and results of the linear mixed models on communication strategy use (Communication Profile for the Hearing Impaired; primary outcome).

Communication strategy use subscales and group				T0				T1				T2				T3				*P* value^a^
				n		mean (SD)		n		mean (SD)		n		mean (SD)		n		mean (SD)		
**Intention to treat**																				
	**Maladaptive behaviors**																		.84	
		SUPR group	167		4.4 (0.5)		149		4.6 (0.4)		130		4.6 (0.4)		128		4.6 (0.4)			
		Control group	152		4.4 (0.6)		142		4.5 (0.5)		129		4.5 (0.6)		126		4.6 (0.5)			
	**Verbal strategies**																		.09	
		SUPR group	167		2.2 (0.6)		149		2.3 (0.7)		130		2.3 (0.7)		128		2.3 (0.7)			
		Control group	152		2.3 (0.7)		142		2.2 (0.7)		129		2.2 (0.7)		126		2.3 (0.7)			
	**Nonverbal strategies**																		.09	
		SUPR group	167		2.9 (0.8)		149		3.0 (0.9)		130		2.9 (0.9)		128		2.9 (0.9)			
		Control group	152		3.0 (1.0)		142		2.9 (0.9)		129		2.9 (0.9)		126		2.9 (0.9)			
**Per protocol**																				
	**Maladaptive behaviors**																		.92	
		SUPR group	60		4.4 (0.5)		56		4.6 (0.4)		54		4.5 (0.5)		46		4.6 (0.4)			
		Control group	151		4.4 (0.6)		141		4.5 (0.5)		128		4.5 (0.6)		125		4.6 (0.5)			
	**Verbal strategies**																		.11	
		SUPR group	60		2.2 (0.6)		56		2.3 (0.8)		54		2.4 (0.9)		46		2.3 (0.8)			
		Control group	151		2.3 (0.7)		141		2.2 (0.7)		128		2.2 (0.7)		125		2.3 (0.7)			
	**Nonverbal strategies**																		.06	
		SUPR group	60		2.8 (0.9)		56		2.9 (0.9)		54		2.9 (1.0)		46		2.9 (0.9)			
		Control group	151		3.1 (1.0)		141		2.9 (0.9)		128		2.9 (0.9)		125		2.9 (0.9)			

^a^*P* value for difference in the course of the outcomes between groups (interaction term time*group). A *P* value of <.016 was considered statistically significant.

#### Secondary Outcomes

##### Intention to Treat Analysis

There were no differences in secondary outcomes between first-time and experienced clients unless stated otherwise. The results for the psychosocial outcomes are presented in Table 4. There were no statistically significant group differences in the course of these outcomes (*P*≥.05).

The results on self-efficacy for HA handling are presented in Multimedia Appendix 1. A statistically significant (*P*<.05) group difference in self-efficacy for basic HA handling over time (time*group: *P*=.01) was observed. However, post hoc analyses showed no statistically significant (*P*≥.016) differences at the follow-up measurements. Immediately postintervention, the self-efficacy for advanced HA handling scores were significantly (*P*<.05) higher for the SUPR group than for the control group (5.3, 95% CI 0.3-10.4; *P*=.04). This effect was sustained at 6- and 12-month follow-up (time*group *P*=.56, so *P*≥.05).

Multimedia Appendix 2 shows the results for HA use. Immediately postintervention, the SUPR group had significantly (*P*<.05) greater HA use compared with the controls (mean difference 0.3, 95% CI 0.02 to 0.5; *P*=.03). This group difference was not maintained at 6- and 12-month follow-up (time*group *P*=.008, so *P*<.05). There was a statistically significant group difference neither immediately postintervention nor in the course of the outcome HA use pattern.

The results of the IOI-HA and IOI-AI item scores are also presented in Multimedia Appendix 2. Note that the IOI-HA items on satisfaction and quality of life were assessed both for SUPR and control groups. Immediately postintervention, HA satisfaction was significantly (*P*<.05) greater in the SUPR group than in the controls (mean difference 0.3, 95% CI 0.09 to 0.5; *P*=.006). This group difference was maintained at 6- and 12-month follow-up (ie, time*group *P*=.05, so *P*≥.05). There was no significant group difference in the course of quality of life. When examining the IOI outcomes in which HAs were directly compared with SUPR, there were significant (*P*<.05) group differences in favor of the controls immediately postintervention on satisfaction with the intervention (mean difference –0.6, 95% CI –0.9 to –0.4; *P*<.001), benefit experienced from the intervention (mean difference –1.0, 95% CI –1.3 to –0.8; *P*<.001), and quality of life (mean difference –0.4, 95% CI –0.7 to –0.2; *P*<.001). These effects were maintained at 6- and 12-month follow-up (time*group *P*=.30, time*group *P*=.24, and time*group *P*=.41, respectively, so *P*≥.05). In other words, controls experienced greater levels of satisfaction, benefit, and quality of life resulting from their HAs than the SUPR recipients did with the SUPR program. There were no significant group differences when comparing the IOI-HA and IOI-AI item residual activity limitations, residual participation restrictions, and impact on others.

**Table 4:** Descriptive statistics and results of the linear mixed models on personal adjustment (Communication Profile for the Hearing Impaired) and emotional response (Hearing Handicap and Disability Inventory; secondary outcomes).

Psychosocial outcomes and group			T0			T1			T2		T3			*P* value^a^
			n	Mean (SD)	n		Mean (SD)	n		Mean (SD)	n	Mean (SD)		
**Personal adjustment to hearing loss**														
	**Self-acceptance**												.63	
		SUPR group	167	4.2 (0.7)	149		4.4 (0.6)	129		4.4 (0.7)	128	4.5 (0.5)		
		Control group	152	4.2 (0.8)	142		4.4 (0.7)	128		4.4 (0.6)	126	4.4 (0.7)		
	**Acceptance of loss**												.12	
		SUPR group	167	3.6 (0.7)	149		3.9 (0.8)	129		3.8 (0.8)	128	3.8 (0.8)		
		Control group	152	3.5 (0.8)	142		3.9 (0.8)	128		3.8 (0.9)	126	4.0 (0.8)		
	**Stress and withdrawal**												.89	
		SUPR group	167	3.5 (0.7)	149		3.9 (0.7)	129		3.8 (0.8)	128	3.8 (0.8)		
		Control group	152	3.5 (0.9)	142		3.9 (0.8)	128		3.9 (0.8)	126	3.9 (0.8)		
**Emotional response**													.36	
	SUPR group		167	1.3 (0.7)	149		0.9 (0.7)	127		1.0 (0.7)	128	1.0 (0.7)		
	Control group		151	1.4 (0.8)	140		1.0 (0.8)	126		1.0 (0.7)	124	1.0 (0.7)		

^a^*P* value for difference in the course of the outcomes between groups (interaction term time*group). A *P* value of <.05 was considered statistically significant.

Multimedia Appendix 3 displays the results on recommendation of the practice services, readiness to act on hearing loss, and self-reported hearing disability. A significant (*P*<.05) group difference over the course of the URICA-HL action score (time*group: *P*=.01) was found. However, the post hoc analyses indicated no statistically significant (*P*≥.016) group differences at the follow-up measurements. A significant (*P*<.05) interaction for type of HA client was found for the URICA-HL committed action score (*P*=.03). A significant (*P*<.05) group difference was found for experienced clients (time*group *P*=.001), while for first time clients there was no difference (*P*=.46). However, post hoc analyses showed no statistically significant (*P*≥.016) group differences for the experienced clients on the committed action scores at the follow-up measurements. No significant group differences were observed for the other URICA scales or self-reported hearing disability.

##### Per Protocol Analysis

In total, 35.0% (63/180) of clients in the SUPR group were included in the per protocol analysis as they had clicked through to a video on communication strategies and personal adjustment from at least two emails. Almost all control clients (98.8%) (161/163) were included in the per protocol analysis; only 2 controls had received SUPR (due to an administrative error). Differences between ITT and per protocol analysis were observed for some outcomes. First, contrary to the significantly better self-efficacy for advanced HA handling and HA use observed for the SUPR group in the ITT analysis, the per protocol analysis did not reveal any significant (*P*≥.05) group differences immediately postintervention (*P*=.23 and *P*=.052, respectively). Second, contrary to the ITT analysis, there was no significant (*P*≥.05) difference in the course of the action scores (time*group *P*=.21). Next, contrary to the absence of a difference on HA use pattern found in the ITT analysis, the per protocol analysis showed a significant (*P*<.05) group difference immediately postintervention, such that the SUPR group had a more stable pattern in HA use than controls (–0.4, 95% CI –0.7 to 0.04; *P*=.03; a lower score indicated a more stable pattern in HA use). This effect was sustained at 6- and 12-month follow-up (time*group *P*=.92, so *P*≥.05). Fourth, contrary to the ITT analysis, there was no significant (*P*≥.05) group difference in quality of life resulting from using the intervention immediately postintervention (*P*=.052). Last, similar to the ITT analysis, the significant group difference on satisfaction with the intervention and benefit experienced from the intervention was also found in the per protocol analysis. However, contrary to the ITT analysis, the per protocol analysis showed statistically significant (*P*<.05) different effects for first-time and experienced clients. For both outcomes, a long-term effect was observed for experienced clients (similar to the ITT analysis). For new clients, the effect was only observed at 6- and 12-month follow-up and not immediately postintervention.

## Discussion

### Principal Findings

Using a cRCT design, we evaluated the effectiveness of SUPR, a web-based self-management support program provided as an addition to regular HA fitting to HA users aged 50 years and over in order to improve the self-management of hearing difficulties and HA use. The study showed that SUPR did not lead to more frequent use of communication strategies (primary outcome) compared with care as usual. Nonetheless, SUPR significantly improved clients’ self-efficacy for advanced HA handling and HA satisfaction at 12 months, as well as HA use immediately postintervention. No group differences were observed for any of the remaining secondary outcomes.

It is encouraging to see that SUPR was able to significantly increase HA use immediately postintervention. Although the effect seems small (mean difference of 0.3 on a scale from 1 to 5), the fact that SUPR was able to improve HA use can be considered valuable because a recently published systematic review indicated that there is no evidence of interventions showing any improvements on HA use on the short, medium, or long term [10]. The evidence was judged as limited because the majority of previous studies had nonpowered small sample sizes (limiting the occurrence of statistically significant differences) or were carried out in nonrepresentative samples (ie, military veteran populations). The SUPR study can thus be regarded as an important addition to the existing body of evidence. Moreover, the per protocol analysis revealed a significant positive effect of SUPR on HA use stability in the long term, indicating a more stable HA use pattern (ie, the same number of hours of HA use every day) among SUPR participants than among controls. The absence of a long-term improvement in HA use may imply that without support like SUPR, HA clients may tend to fall back and be left to full self-management. This underlines the importance of having follow-up support after clients have completed SUPR to increase the likelihood of extending the effects to the long term.

It has been argued that more frequent or more stable HA use does not automatically imply more satisfaction with HAs [56] and that HA use (in hours) alone cannot be viewed as an indicator of successful HA use [57]. From this perspective, it is interesting to see that we not only found increased HA use in SUPR recipients, but also significantly greater HA satisfaction than the controls postintervention. This shows that the increase in hours of use indeed coincided with greater HA satisfaction.

This study demonstrated that the SUPR group had a significantly higher score of 5.3 points (95% CI 0.3 to 10.4; scored on a scale from 0 to 100) on the advanced HA handling self-efficacy scale directly postintervention than controls that lasted up to 12 months later. Both groups seemed to perform at ceiling on the basic HA handling self-efficacy scale, suggesting that during appointments dispensers already ensured clients became skilled in basic HA handling (ie, HA and battery insertion and removal, HA cleaning and maintenance). This was also stated by Ferguson et al [32], who found similar ceiling effects. Although the instruction videos mostly focused on basic rather than advanced HA handling skills, watching them might have increased clients’ confidence in their ability to also handle more advanced skills, like troubleshooting or naming the model of a particular HA. We are uncertain about the clinical meaning of a difference of 5.3 points, however. Further research should address this.

Bennett et al [58] found that most problems HA owners experienced were related to HA management including HA use, handling, and ongoing care and that these had the greatest impact on HA success. SUPR improving HA clients’ confidence in their ability to manage their HAs can thus be considered an encouraging finding. Other work by Bennett et al [59] showed that HA management can be classified according to two overarching themes: the device and the person. The results of our study suggest that SUPR is successful in improving HA management concepts related to the device (HA maintenance and repairs, daily HA use, advanced HA knowledge) but not for HA management related to the person (learning to come to terms with HAs, communication strategies, working with a clinician). This is further discussed below.

Previous studies have shown that both individual and group auditory rehabilitation interventions can effectively increase communication strategy use [22,31,33,60] both in the short- and long term. A plausible explanation for the absence of an effect in this study may be related to the video-watching rates as reported in our process evaluation study (submitted). Whereas up to 37% of the participants in the intervention arm had watched the instruction videos, only 7% to 16% had watched the videos on communication strategies and personal adjustment. Not engaging with web-based interventions as was intended is a well-known problem in intervention effectiveness research [61,62]. The per protocol analysis still showed no effects on communication strategy use, but it must be noted that the sensitivity analysis was most likely underpowered (SUPR n=60, care as usual n=151). We intended to perform the per protocol analysis with clients who had clicked on more than two video links, but this resulted in samples too small to allow a meaningful statistical analysis.

An alternative explanation for the absence of a communication strategy use or personal adjustment effect may be related to the setting in which SUPR was provided. Given that the HA dispensing setting is a primary care one, mainly focusing on HA fitting and dispensing, clients may not have expected or been ready for receiving educational videos. Such type of rehabilitation may better fit in specialized (ie secondary or tertiary) hearing health care settings. Kramer et al [33] chose a tertiary setting to provide their home education program, resulting in improved communication strategy skills.

Control group participants reported significantly greater benefit, satisfaction, and quality of life because of their HAs (IOI-HA) than SUPR participants reported for SUPR (IOI-AI). This suggests that HAs were viewed as more impactful than SUPR. In a way, this is not surprising, since HAs can be considered as a basis. They amplify sounds and may thus improve listening ability and thereby quality of life [63]. It is the combination of the two (HAs and additional support) that may be most beneficial [10]. Also, it is important to consider that HAs have a long history (more than a century) of development while the development of (web-based) support programs is still in its infancy. There is much to learn still to further refine communication programs to ensure they fit clients’ needs and have a larger impact.

The URICA questionnaire outcomes conflicted with what was expected both in the direction of the effect and changes over time and were therefore difficult to interpret. We expected the SUPR group to show an increase in the action scores, while in fact the follow-up scores were lower than baseline in both groups. Differences in interpretation of what taking action meant to someone may have occurred between participants but also within participants over time (as the intervention might have influenced what taking action was) causing invalid measurements. This is further discussed in Meijerink et al [40].

### Strengths and Limitations

This was the first audiological rehabilitation study implementing and evaluating a web-based self-management support program on such a large scale and in a real-life HA dispensing setting. The large sample size, use of a robust RCT design, and outcome assessment at the short, medium, and long term can be considered unique in our research field. Given these strengths, the study tackles most of the limitations mentioned in a recent meta-analysis on intervention studies to improve HA use [10]. Including 70 clusters across the Netherlands and purposefully sampling for spread in degree of rural/urban areas minimized imbalance across treatment groups and increased the generalizability of the findings to the Dutch real-world practice. Nevertheless, there are a number of limitations.

First, while we did reach our targeted sample size for the first-time clients, we did not for the experienced clients due to recruitment difficulties. The limited number of experienced clients may have resulted in nonsignificant differences in effects between first-time and experienced clients. A second limitation is that clients had to provide consent for study participation while knowing their group allocation. This may have affected clients’ willingness to participate in the study and introduced selection bias. Unfortunately, it was not possible to prevent this because randomizing the practices after obtaining clients’ consent would have delayed the intervention period by 7 months until clients had provided their consent. This was deemed unacceptable given the real-life character of the study. A third limitation was that participating clients, dispensers and researchers could not be blinded. This may have introduced bias. Possibly, clients who were aware of receiving SUPR may have responded more favorably compared to controls, while controls being aware of receiving standard care only may have sought alternative treatments (which would have increased the likelihood of contamination). We attempted, however, to prevent controls from seeking access to SUPR by reducing the amount of information given about SUPR content and by offering controls SUPR after study completion. A fourth limitation is that we initially aimed to measure HA use using data logging in order to measure HA use objectively, but this appeared unfeasible. Hence, all outcomes were self-reported, possibly resulting in overreporting of HA use [18,48]. Finally, behavior change was expected at multiple levels, and therefore many outcomes were evaluated. This increased the likelihood of finding statistically significant results by chance. We therefore applied Bonferroni corrections for the primary outcomes and the post hoc analyses but not for the secondary outcomes. It should be noted that there is debate among statisticians as to when multiple outcomes should be corrected for [64,65]. Using a Bonferroni correction for all outcomes is concerned too strict by some as it would increase the chance for false negatives [66].

### Conclusions

While the popularity of web-based platforms to complement HA fitting is rising [30-32], high-quality evidence (ie, assuring external validity and power) to show the long-term benefits of eHealth in HA rehabilitation is still lacking [37]. This study is a valuable addition to the existing evidence for such platforms in hearing health care. While SUPR did not significantly enhance the use of communication strategies, this study provides evidence for the effectiveness of SUPR to improve self-efficacy for HA handling and HA satisfaction in the long term and HA use in the short term. Given that the effects were mainly found in the HA handling domain, this study indicates that an intervention offering web-based instructions is a promising addition to the services provided by dispensers. Further research is needed to evaluate if adjustments to SUPR will lead to a higher adherence of clients in following the intervention to improve the (long-term) effectiveness of communication strategy use and other psychosocial outcomes.

## Appendix

Multimedia Appendix 1 Descriptive statistics and results of the linear mixed models on self-efficacy for hearing aid handling (Measure of Audiologic Rehabilitation Self-Efficacy for Hearing Aids; secondary outcome).

Multimedia Appendix 2 Descriptive statistics and results of the linear mixed models on hearing aid use (International Outcome Inventory for Hearing Aids) and hearing aid pattern (use questionnaire; secondary outcomes).

Multimedia Appendix 3 Descriptive statistics and results of the linear mixed models on recommendation of the services of the hearing aid dispensing practice, readiness to act on hearing loss (University of Rhode Island Change Assessment adapted for hearing loss), and self-reported hearing disability (Amsterdam Inventory for Auditory Disability and Handicap; secondary outcomes).

## Abbreviations

AIADH: Amsterdam Inventory for Auditory Disability and Handicap
CONSORT: Consolidated Standards of Reporting Trials
cRCT: cluster randomized controlled trial
CPHI: Communication Profile for the Hearing Impaired
HA: hearing aid
HHDI: Hearing Handicap and Disability Inventory
ICC: intracluster correlation coefficient
IOI: International Outcome Inventory
IOI-AI: International Outcome Inventory for Alternative Interventions
IOI-HA: International Outcome Inventory for Hearing Aids
ITT: intention to treat
MARS-HA: Measure of Audiologic Rehabilitation Self-Efficacy for Hearing Aids
URICA-HL: University of Rhode Island Change Assessment adapted for hearing loss
