# Supplementary material for: Effectiveness of a Web-Based SUpport PRogram (SUPR) for Hearing Aid Users Aged 50+: Two-Arm, Cluster Randomized Controlled Trial
Source: J Med Internet Res. 2020 Sep 22;22(9):e17927. doi: 10.2196/17927 (PMC7539169; doi:10.2196/17927)
Supplement: Multimedia Appendix 1 [file jmir_v22i9e17927_app1.docx]

| Multimedia Appendix 1. Descriptive statistics and results of the linear mixed models on self-efficacy for hearing aid handling (MARS-HA) (secondary outcome). | | | | | | | | | |  |
| --- | --- | --- | --- | --- | --- | --- | --- | --- | --- | --- |
|  | |  | T1 |  | T2 |  | T3 |  | LMM^a^ | |
|  | | Group | n | Mean (SD^b^) | n | Mean (SD) | n | Mean (SD) | *P^c^* | |
| **Self-efficacy for HA^d^ handling** | |  |  |  |  |  |  |  |  | |
|  | Basic | SUPR group | 149 | 96.3 (7.0) | 129 | 96.8 (5.7) | 128 | 97 (5.2) | .01 | |
|  |  | Control group | 142 | 96.3 (7.2) | 128 | 95.3 (9.6) | 125 | 97.1 (6.3) |  | |
|  | Advanced | SUPR group | 149 | 71.8 (22.1) | 129 | 72.4 (20.9) | 128 | 71.5 (20.7) | .56 | |
|  |  | Control group | 142 | 67.2 (23.1) | 128 | 66.2 (23.7) | 125 | 67 (22.5) |  | |
|  | |  | MD^e^  (95% CI^h^) | *P*^f^ | MD  (95% CI) | *P*^g^ | MD  (95% CI) | *P*^g^ |  | |
| Basic | |  | -0.01  (-1.7-1.6) | .99 | 2.0  (0.3-3.7) | .02 | 0.07  (-1.6-1.8) | .92 |  | |
| Advanced | |  | 5.3  (0.3-10.4) | .04 |  |  |  |  |  | |

^a^LMM: linear mixed models.

^b^SD: standard deviation.

^c^*P* value for difference in in the course of the outcomes between groups (interaction term time*group). A *P value* of <.05 was considered statistically significant.

^d^HA: hearing aid.

^e^MD: mean difference. A positive mean difference indicates a difference (ie a higher score) in favor of the intervention group compared with the control group.

^f^*P* value for difference between mean values in SUPR and control group immediately post-intervention (t1). A *P* value of <.05 was considered statistically significant.

^g^*P* value for difference between mean values in SUPR and control group at six and twelve months follow-up (post-hoc analyses). Note that these are only indicated in case of a significant interaction term (time*group). For post-hoc analyses, a *P value* of <.016 was considered statistically significant.

^h^CI: confidence interval.
