# Supplementary material for: Effectiveness of a Web-Based SUpport PRogram (SUPR) for Hearing Aid Users Aged 50+: Two-Arm, Cluster Randomized Controlled Trial
Source: J Med Internet Res. 2020 Sep 22;22(9):e17927. doi: 10.2196/17927 (PMC7539169; doi:10.2196/17927)
Supplement: Multimedia Appendix 2 [file jmir_v22i9e17927_app2.docx]

| Multimedia Appendix 2. Descriptive statistics and results of the linear mixed models on hearing aid use (IOI-HA) and hearing aid pattern (use-questionnaire) (secondary outcomes). | | | | | | | |  |  |
| --- | --- | --- | --- | --- | --- | --- | --- | --- | --- |
|  |  | T1 |  | T2 |  | T3 |  | | LMM^a^ |
|  | Group | n | Mean (SD^b^) | n | Mean (SD) | n | Mean (SD) | | *P^c^* |
| HA^d^ use | SUPR group | 149 | 4.6 (0.8) | 129 | 4.4 (1.2) | 128 | 4.4 (1.1) | | .008 |
|  | Control group | 142 | 4.4 (1.1) | 127 | 4.3 (1.0) | 125 | 4.4 (1.0) | |  |
| HA use pattern | SUPR group | 149 | 1.8 (1.1) | 129 | 1.8 (1.1) | 128 | 1.9 (1.1) | | .51 |
|  | Control group | 142 | 2.0 (1.1) | 127 | 2.0 (1.1) | 125 | 2.0 (1.1) | |  |
| Satisfaction with HA | SUPR group | 149 | 4.1 (0.9) | 129 | 3.9 (0.9) | 128 | 3.9 (1.0) | | .050 |
|  | Control group | 142 | 3.8 (1.0) | 127 | 3.8 (1.0) | 125 | 3.8 (1.0) | |  |
| Quality of life with HA | SUPR group | 149 | 4.0 (0.9) | 129 | 3.8 (1.0) | 128 | 3.8 (1.1) | | .39 |
|  | Control group | 142 | 3.9 (0.9) | 127 | 3.8 (1.0) | 125 | 3.8 (1.0) | |  |
| Benefit of HA or SUPR | SUPR group | 149 | 2.8 (1.3) | 129 | 2.7 (1.2) | 128 | 2.4 (1.3) | | .24 |
|  | Control group | 142 | 3.8 (0.9) | 127 | 3.7 (1.1) | 125 | 3.7 (1.0) | |  |
| Residual activity limitations with HA or SUPR | SUPR group | 149 | 4.0 (1.0) | 129 | 4.1 (0.9) | 128 | 4.1 (1.1) | | .44 |
|  | Control group | 142 | 4.0 (0.8) | 127 | 4.0 (0.8) | 125 | 4.1 (0.9) | |  |
| Satisfaction with HA or SUPR | SUPR group | 149 | 3.1 (1.1) | 129 | 3.0 (1.1) | 128 | 3.0 (1.2) | | .30 |
|  | Control group | 142 | 3.8 (1.0) | 127 | 3.8 (1.0) | 125 | 3.8 (1.0) | |  |
| Residual participation restrictions with HA or SUPR | SUPR group | 149 | 4.3 (0.9) | 129 | 4.3 (1.0) | 128 | 4.3 (1.0) | | .79 |
|  | Control group | 142 | 4.3 (0.8) | 127 | 4.3 (0.7) | 125 | 4.4 (0.7) | |  |
| Impact on others with HA or SUPR | SUPR group | 149 | 4.4 (0.8) | 129 | 4.3 (0.9) | 128 | 4.4 (0.8) | | .38 |
|  | Control group | 142 | 4.5 (0.7) | 127 | 4.4 (0.7) | 125 | 4.4 (0.7) | |  |
| Quality of Life with HA or SUPR | SUPR group | 149 | 3.5 (1.1) | 129 | 3.3 (1.0) | 128 | 3.2 (1.1) | | .41 |
|  | Control group | 142 | 3.9 (0.9) | 127 | 3.8 (1.0) | 125 | 3.8 (1.0) | |  |
|  |  | MD^e^  (95% CI^h^) | *P*^f^ | MD  (95% CI) | *P*^g^ | MD  (95% CI) | *P*^g^ | |  |
| HA use |  | 0.3  (0.02–0.5) | .03 | 0.05  (-0.2–0.3) | .64 | -0.0001  (-0.2–0.2) | .99 | |  |
| HA use pattern |  |  | .06 |  |  |  |  | |  |
| Satisfaction with HA |  | 0.3  (0.09–0.5) | .006 |  |  |  |  | |  |
| Quality of life with HA |  |  | 0.34 |  |  |  |  | |  |
| Benefit of HA or SUPR |  | -1.0  (-1.3–-0.8) | <.001 |  |  |  |  | |  |
| Residual activity limitations with HA or SUPR |  |  | 0.78 |  |  |  |  | |  |
| Satisfaction with HA or SUPR |  | -0.6  (-0.9–-0.4) | <.001 |  |  |  |  | |  |
| Residual participation restrictions with HA or SUPR |  |  | 0.86 |  |  |  |  | |  |
| Impact on others with HA or SUPR |  |  | 0.44 |  |  |  |  | |  |
| Quality of Life with HA or SUPR |  | -0.4  (-0.7–-0.2) | <.001 |  |  |  |  | |  |

^a^LMM: linear mixed models.

^b^SD: standard deviation.

^c^*P* value for difference in in the course of the outcomes between groups (interaction term time*group). A *P value* of <.05 was considered statistically significant.

^d^HA: hearing aid.

^e^MD: mean difference. A positive mean difference indicates a difference (ie a higher score) in favor of the intervention group compared with the control group.

^f^*P* value for difference between mean values in SUPR and control group immediately post-intervention (t1). A *P* value of <.05 was considered statistically significant.

^g^*P* value for difference between mean values in SUPR and control group at six and twelve months follow-up (post-hoc analyses). Note that these are only indicated in case of a significant interaction term (time*group). For post-hoc analyses, a *P* value of <.016 was considered statistically significant.

^h^CI: confidence interval.
