# Supplementary material for: Effectiveness of a Web-Based SUpport PRogram (SUPR) for Hearing Aid Users Aged 50+: Two-Arm, Cluster Randomized Controlled Trial
Source: J Med Internet Res. 2020 Sep 22;22(9):e17927. doi: 10.2196/17927 (PMC7539169; doi:10.2196/17927)
Supplement: Multimedia Appendix 3 [file jmir_v22i9e17927_app3.docx]

| Multimedia Appendix 3. Descriptive statistics and results of the linear mixed models on recommendation of the services of the HA dispensing practice, readiness to act on hearing loss (URICA-HL), and self-reported hearing disability (AIADH) (secondary outcomes). | | | | | | | | | | | | | | | | | | | |  |  |
| --- | --- | --- | --- | --- | --- | --- | --- | --- | --- | --- | --- | --- | --- | --- | --- | --- | --- | --- | --- | --- | --- |
|  | | |  | T0 |  | T1 |  |  |  | T2 |  |  |  | T3 |  |  |  | LMM^a^ | | |  |
|  | | | Group | n | Mean (SD^b^) | n | Mean (SD) | MD^c^  (95% CI^d^) | *P*^e^ | n | Mean (SD) | MD  (95% CI) | *P*^f^ | n | Mean (SD) | MD  (95% CI) | *P*^f^ | *P*^g^ | | |  |
| **Recommendation of the services of the HA^h^ dispensing practice** | | | SUPR group | 167 | 8.5 (1.8) | 149 | 8.6 (1.7) |  |  | 129 | 8.2 (1.9) |  |  | 128 | 8.2 (1.8) |  |  | .98 | | |  |
|  | | | Control group | 152 | 8.3 (2.1) | 142 | 8.4 (2.1) |  |  | 127 | 8.1 (2.1) |  |  | 125 | 8.0 (2.4) |  |  |  | | |  |
| **Readiness to act on hearing loss** | | |  |  |  |  |  |  |  |  |  |  |  |  |  |  |  |  | | |  |
|  | Precontemplation | | SUPR group | 167 | 1.8 (0.6) | 149 | 2.3 (0.7) |  |  | 127 | 2.4 (0.6) |  |  | 128 | 2.2 (0.6) |  |  | | .12 | | |
|  |  | | Control group | 151 | 1.9 (0.6) | 140 | 2.2 (0.7) |  |  | 126 | 2.2 (0.6) |  |  | 124 | 2.2 (0.6) |  |  | |  | | |
|  | Contemplation | | SUPR group | 167 | 4.2 (0.7) | 149 | 3.5 (0.7) |  |  | 129 | 3.4 (0.8) |  |  | 128 | 3.4 (0.8) |  |  | | .82 | | |
|  |  | | Control group | 151 | 4.3 (0.8) | 141 | 3.5 (0.9) |  |  | 127 | 3.4 (0.9) |  |  | 125 | 3.4 (0.9) |  |  | |  | | |
|  | Preparation | | SUPR group | 167 | 3.8 (0.8) | 149 | 3.0 (0.9) |  |  | 128 | 2.8 (0.9) |  |  | 128 | 2.9 (0.9) |  |  | | .32 | | |
|  |  | | Control group | 151 | 3.6 (0.9) | 140 | 2.9 (1.0) |  |  | 126 | 2.8 (1.0) |  |  | 124 | 2.9 (0.9) |  |  | |  | | |
|  | Action | | SUPR group | 167 | 4.2 (0.6) | 149 | 3.5 (0.8) | 0.2  (0.02-0.4) | 0.03 | 127 | 3.3 (0.8) | -0.05  (-0.2-0.1) | 0.58 | 128 | 3.2 (0.9) | -0.1  (-0.3-0.1) | 0.34 | | .010 | | |
|  |  | | Control group | 151 | 4.2 (0.7) | 140 | 3.3 (0.8) |  |  | 126 | 3.3 (0.8) |  |  | 124 | 3.3 (0.8) |  |  | |  | | |
|  | Maintenance | | SUPR group | - | - | 149 | 2.4 (0.8) |  | 0.69 | 127 | 2.4 (0.8) |  |  | 128 | 2.4 (0.8) |  |  | | .58 | | |
|  |  | | Control group | - | - | 140 | 2.4 (0.9) |  |  | 126 | 2.5 (0.9) |  |  | 124 | 2.4 (0.8) |  |  | |  | | |
|  | Readiness | | SUPR group | - | - | 149 | 54.6 (17.8) |  | 0.33 | 127 | 50.8 (17.7) |  |  | 128 | 51.8 (18.7) |  |  | | .06 | | |
|  |  | | Control group | - | - | 140 | 52.4 (18.2) |  |  | 126 | 52.8 (17.1) |  |  | 125 | 52.7 (17.9) |  |  | |  | | |
|  | Committed action – first-time clients | | SUPR group | 108 | 21.6 (4.0) | 94 | 16.6 (5.6) |  |  | 83 | 16.4 (5.5) |  |  | 82 | 16.1 (5.7) |  |  | | .46 | | |
|  |  | | Control group | 95 | 20.1 (4.8) | 90 | 15.7 (5.4) |  |  | 83 | 15.8 (5.5) |  |  | 82 | 15.8 (5.4) |  |  | |  | | |
|  | Committed action – experienced clients | | SUPR group | 59 | 20.4 (5.0) | 55 | 18.1 (5.5) | 2.4  (0.3-4.4) | 0.02 | 44 | 15.5 (5.1) | -1.8  (-4.0-0.3) | 0.10 | 128 | 14.9 (5.7) | -2.0  (-4.2-0.2) | 0.072 | | .001 | | |
|  |  | | Control group | 56 | 20.7 (5.0) | 50 | 15.6 (5.6) |  |  | 43 | 17.2 (5.1) |  |  | 124 | 16.9 (5.7) |  |  | |  | | |
| **Self-reported hearing disability** | | |  |  |  |  |  |  |  |  |  |  |  |  |  |  |  |  | | |  |
|  | | Distinction of sounds | SUPR group | 166 | 8.0 (4.6) | 150 | 4.7 (3.6) |  |  | 130 | 5.3 (4.4) |  |  | 128 | 4.8 (3.9) |  |  | | .10 | | |
|  | |  | Control group | 152 | 8.3 (4.3) | 142 | 4.4 (3.7) |  |  | 129 | 4.6 (3.9) |  |  | 127 | 4.7 (4.1) |  |  | |  | | |
|  | | Auditory localization | SUPR group | 160 | 6.9 (3.6) | 151 | 4.1 (3.0) |  |  | 130 | 4.2 (3.3) |  |  | 128 | 4.4 (3.3) |  |  | | .40 | | |
|  | |  | Control group | 153 | 7.0 (3.3) | 142 | 4.2 (3.2) |  |  | 129 | 4.1 (3.4) |  |  | 127 | 3.9 (3.3) |  |  | |  | | |
|  | | Intelligibility in noise | SUPR group | 174 | 9.5 (2.8) | 151 | 5.5 (3.1) |  |  | 130 | 6.1 (3.1) |  |  | 128 | 5.9 (3.1) |  |  | | .41 | | |
|  | |  | Control group | 157 | 9.5 (3.0) | 142 | 5.5 (3.4) |  |  | 129 | 5.8 (3.6) |  |  | 127 | 5.7 (3.4) |  |  | |  | | |
|  | | Intelligibility in quiet | SUPR group | 170 | 77 (2.9) | 151 | 4.4 (2.7) |  |  | 131 | 4.8 (3.0) |  |  | 128 | 4.7 (3.1) |  |  | | .06 | | |
|  | |  | Control group | 155 | 7.7 (3.0) | 144 | 4.1 (3.1) |  |  | 129 | 4.0 (3.1) |  |  | 127 | 4.2 (3.1) |  |  | |  | | |
|  | | Detection of sounds | SUPR group | 174 | 5.6 (2.8) | 150 | 2.9 (2.2) |  |  | 130 | 3.0 (2.3) |  |  | 128 | 3.1 (2.3) |  |  | | .42 | | |
|  | |  | Control group | 155 | 5.8 (2.9) | 142 | 2.9 (2.4) |  |  | 129 | 2.9 (2.5) |  |  | 127 | 3.0 (2.5) |  |  | |  | | |

^a^LMM: linear mixed models.

^b^SD: standard deviation.

^c^MD: mean difference. A positive mean difference indicates a difference in favor of the intervention group (ie a higher score) compared with the control group.

^d^CI: confidence interval.

^e^*P* value for difference between mean values in SUPR and control group directly post-intervention (t1). A *P* value of <.05 was considered statistically significant.

^f^*P* value for difference between mean values in SUPR and control group at six and twelve months follow-up (post-hoc analyses). Note that these are only indicated in case of a significant interaction term (time*group), see below. For post-hoc analyses, a *P* value of <.016 was considered statistically significant.

^g^*P* value for difference in in the course of the outcomes between groups (interaction term time*group). A *P* value of <.05 was considered statistically significant.

^h^HA: hearing aid.
